# Performance aware shared memory hierarchy model for multicore processors

**DOI:** 10.1038/s41598-023-34297-3

**Published:** 2023-05-05

**Authors:** Ahmed M. Mohamed, Nada Mubark, Saad Zagloul

**Affiliations:** 1 Electrical Engineering Department, Faculty of Engineering, Aswan University, Aswan, Egypt; 2 Computer Science Department, Faculty of Computers and Information, South Valley University, Qena, Egypt; 3 Mathematic Department, Faculty of Science, South Valley University, Qena, Egypt

**Keywords:** Engineering, Mathematics and computing, Applied mathematics, Computer science

## Abstract

Despite the fact that multicore processors have a better instruction execution speed and lower power consumption, they also encounter a set of design challenges. The appearance of multicore and many core architectures has raised the problem of managing shared hierarchical memory systems. The main focus of this paper is to evaluate the behavior of shared hierarchical memory systems by modeling their response time analytically. Since the gap between the memory and processor speed increases rapidly, it gets more crucial to find an analytical model that includes the significant factors that affect the performance of hierarchical memory systems. The proposed model considers the interdependence between different memory layers and differentiates between the memory response time and memory system time. Moreover, the model evaluates the effect of memory hierarchy on the variance of the memory access time. The existence of a large variance can lead to extremely long wait queues which can dramatically affect the performance of multicore processors

## Introduction

Performance modeling plays an essential role in processor design. It can help in determining the architectural parameters that are crucial for optimal performance. Earlier researchers used simulations to evaluate their designs^1^. Nevertheless, simulations may become very time consuming due to the complexities of architectural design spaces and workloads. In modern technology, there is a growing need for high performance computing. As Moore’s Law continues to hold, we will be able to pack even more transistors on a single die. But what happens if this trend continues? On the positive side, we have the advantage to enhance the performance of multicore processors by increasing the number of cores. Multicore processors consume less power and generate less heat per core than the same number of single-core processors.

Multicore processors use shared memory hierarchies to achieve a high-speed memory system. One important characteristic of multicore processors is that they have a different degree of memory system sharing at different levels of the memory hierarchy. Most multicore processors' have cores with a private L1 cache. According to the architecture, an L2 cache may be shared by two or more cores; and an L3 cache is shared as well. The main memory level is shared among all cores of the processor. The memory sharing may vary from one multicore processor to another. While the performance of memory hierarchies is essential to single core processors, it is even more critical to multicore processors. Due to this memory sharing, there is a possibility that for memory intensive applications to occupy the shared memory system leading to a degraded performance. Contention for shared memory hierarchies may be so critical that the only way to run a task may leave many cores idle^1,2^. Although there are many improvements in memory system performance but still there is a significant gap between memory system speed and processor speed. Even if the new multicore processors use a faster memory, there will still be a chance for contention as long as the memory system is shared among the cores. As the number of cores increases, the performance of more applications will be significantly affected by the memory hierarchy contention^3,4^.

In this paper, we propose an analytical model for memory hierarchy systems that takes into account the essential parameters that affect the performance of memory systems. These parameters include the number of layers, the hit ratio of each layer, the access time of each layer, the search time in each layer, the arrival distribution of memory requests and the service distribution of memory requests. We use Markov chains and the M/G/1 queuing model to estimate the average and the variance of the response time for hierarchical memory systems. We use the Linear Algebraic queueing Theory (LAQT) to be able to model deep memory hierarchies. The proposed model can be used to estimate the impact of the number of levels of the memory hierarchy on the variance of the response time. Large variance can produce long wait queues for shared memory systems which can significantly degrade the performance of multicore processors. The rest of the paper is organized as follows, in "Background and motivation", we explore the previous efforts related to the topic of the paper and explain our motivation. We present the system model for the memory hierarchy in "System model". In "The analytical model", we propose the analytical model. Then, in "Simulation results", we show the simulation results and we conclude in "Conclusion".

## Background and motivation

The previous work in modeling hierarchal memory systems can be organized into three categories. The first one is focused on those models of one memory level in single processor machines. Berg et al.^5^ approximated the level one cache performance analytically using the random replacement policy. Another model that derives stack distance (the number of unique memory objects accessed during a reuse epoch) histogram from reuse distance (the number of unique data elements accessed between any two accesses to the same element) histogram to predict level one LRU cache behavior is proposed^6^. Pan et al.^7^ used Markov chains to estimate the cache memory performance under three different replacement algorithms. Artificial neural networks are used to evaluate the impact of out-of-order executions^8^. The second category focused on those models of hierarchal memory systems in a single processor. Ji et al.^9,10^ created an analytical model to estimate the cache misses' ratios using the level one cache stack distance histogram. In^11^, another analytical model was introduced to estimate the level two cache behavior based on the effect of cache inclusion/exclusion policies.

The third category focused on those models of hierarchal memory systems in multicore processors. Nikolov^12^ presented an analytical model for a bus-based shared memory with only private caches. The model intended to capture the whole range of invalidated cache coherence protocols. The model assumes that the memory hierarchy is not shared and the only sharing exists in the main memory. This model focuses on the influence of cache-coherence protocols on the memory system performance but did not take into account other significant factors such as sharing the memory hierarchy. Taecheol et al.^13^, proposed an analytical model to study the impact of memory size and off-chip bandwidth on the performance of multicore processors. The model assumes that the sharing exists on the last level of cache only. This assumption is also used in^14,15^. Jin et al.^16^ presented an analytical model to estimate the performance of two-level memory systems but we see in their presented model a big inconsistency between the forecasted and measured memory access times. Eklov et al.^17^ presented a model that approximates the shared memory miss rates of co-scheduled tasks on memory hierarchal systems. They considered the locality features of the memory requests for each processor core. Though, they did not take into account the impact of data sharing among different processes, which may lead to significant errors when estimating the effect of data sharing on multi-threaded applications. Jasmine et al.^18^ employed Markov chains to model the stack distance histogram of multi-threaded programs. Wu et al.^19^ presented a model to examine the fluctuation of reuse distance by obtaining the profiling information of concurrent reuse distance (the reuse distance of a data/memory reference when the thread is interfered by references from other threads) in Loop-based parallelism. Balasubramonian et al.^20^ indicated that the memory hierarchy systems that do not meet the applications demands will results in a degradation in the performance of most applications. In^21^, the authors have proposed a data-sharing aware analytical model for estimating the miss rates of multi-level cache hierarchies for multi-core processors. The proposed model can also evaluate coherence misses. The authors claim that the overall average absolute error is 5% in four hardware cores configurations. The authors did not take into account the queueing overhead or the utilization of the memory system. The authors in^22^ introduced an experimental study to the impact of multi-level cache hierarchies on the performance of different architectures. For this study, they performed experiments in the Broadwell CPU and Pascal GPU, using applications from the Rodinia benchmark suite. The experiments showed that the main performance limit is the accesses to main memory. In Pascal architecture, the overall memory utilization rate is directly linked to application performance. In the Broadwell architecture, results showed that it is more important to have a total high hit ratio in the memory hierarchy than simply having a high cache hit rate in upper levels such as L1.

Our motivation can be explained as follows. The hierarchical memory systems were considered by several work but all the previous studies to analyze the performance of memory hierarchal systems were limited. These limitations were due to the dependency of the models on the application type or from the analytical model that cannot model deep hierarchal memory systems. In some of the previous studies, we noticed inconsistency between the values produced by analytically models and the measured values due to ignoring the queueing delay for shared memory systems. Also, previous work did not consider the variance of memory access times and its effect on the performance of shared memory systems. In this paper, we propose an analytical model based on Markov chains and the M/G/1 queueing model. The proposed analytical model can be used to model deep memory hierarchies. Also, the proposed model differentiates between the memory response time and memory system time. Moreover, the model evaluates the effect of memory hierarchy on the variance of the memory access time.

## System model

**Figure 1:**
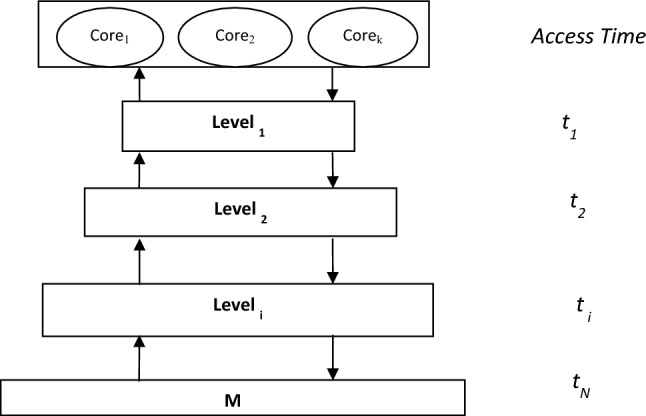
Memory hierarchy system.

We assume that the hierarchical memory system consists of N layers as shown in Fig. 1. The main memory M is the last layer of the hierarchy. Each layer can be accessed by two operations, the fetch operation and the read/write operation. The hit ratio of a memory layer i is h_i_. The average access time of layer i is t_i_ which is divided into two-time parts. The first part is the time taken to search the memory layer for a specific address. The second part is the time taken to read or write the data if found in this layer. We assume that the model uses the write-back protocol. A memory request will be delivered to the cache memory first level. The process of accessing any level of the memory hierarchy starts with searching that level for the required data. The data will be found by probability h then the read/write operation will be performed on that data. The memory request will not be found with probability (1 − h). In this case, the memory request will be forwarded to the next memory level of the hierarchy until we reach the final level of the hierarchy. The required data will be found in the last level with probability one.

## The analytical model

We present two cases of memory use. The first case is the exclusive use of the memory where only one core can access the memory system. The second case is the concurrent access where any number of cores can access the memory system at the same time. If the memory system is idle the request will be handled immediately. Otherwise the request will wait in a FIFO queue until it gets its turn.

### Exclusive memory access

**Figure 2:**
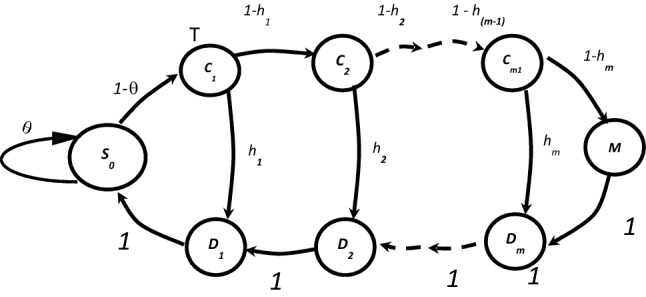
Markov chain for hierarchal memory system.

The memory hierarchy system in Fig. 1 can be modeled by a state diagram as shown in Fig. 2. Each memory layer, i, in Fig. 1 is modeled by two states in Fig. 2. The upper state Ci represents the search process while the lower state Di represents memory read/write process. State S_0_ represents the multicore processor and the last state in the state diagram M is the main memory in the hierarchy. With probability θ the instruction will not need a memory access. We focus on the memory hierarchy only and we will ignore the CPU time and include more details about the memory system to our model. The state diagram in Fig. 2 can be considered as a Markov chain. To estimate the average access time of the deep hierarchal memory system we need to build an analytical model using Linear Algebraic Queuing Technique (LAQT). The main benefit of using LAQT is that we can build our models using matrices. We first need to define the following^23^:

T is a random variable corresponding to the memory hierarchy response time.

P is the transition probability matrix.

p is a vector whose component i is the probability that the memory request starts in state i when it begins using the memory system. The size of row vector the row vector p is n = 2 × N + 1, where N is the number of intermediate levels.

€ is a unit column vector of size n = 2 × N + l.

M is the rate matrix; M is a diagonal matrix where M_i_ = 1/t_i_,

$$\mathrm{P}=\left[\begin{array}{ccccccc}0& 1-{\mathrm{h}}_{1}& 0& \dots \dots \dots & 0& 0& {\mathrm{h}}_{1}\\ 0& 0& 1-{\mathrm{h}}_{2}& \dots \dots \dots & 0& {\mathrm{h}}_{2}& 0\\ .& .& .& .& .& .& .\\ .& .& .& .& .& .& .\\ 0& 0& 0& \dots \dots \dots & 0& 1& 0\\ 0& 0& 0& \dots \dots \dots & 0& 0& 1\\ 0& 0& 0& \dots \dots \dots & 0& 0& 0\end{array}\right]$$

The rows in the matrix P represent the different states in the state diagram. The probability p_*ij*_ is the probability of the system is being in state *i* and moves to state *j*.

$$\mathrm{M}=\left[\begin{array}{cccccccc}\mathrm{M}1& 0& 0& 0& \dots \dots \dots & 0& 0& 0\\ 0& \mathrm{M}2& 0& 0& \dots \dots \dots & 0& 0& 0\\ 0& 0& \mathrm{M}3& 0& \dots \dots \dots & 0& 0& 0\\ .& .& .& .& .& .& .& .\\ .& .& .& .& \mathrm{Mi}& .& .& .\\ 0& 0& 0& 0& \dots \dots \dots & 0& 0& 0\\ 0& 0& 0& 0& \dots \dots \dots & 0& 0& 0\\ 0& 0& 0& 0& \dots \dots \dots & 0& 0& \mathrm{Mn}\end{array}\right]$$

The memory system consists of a set of states as shown in Fig. 2. The memory access time t_i_ at any memory layer can be either exponentially or non-exponentially distributed. For example, for shared memory systems that are using a bus, we can use exponential distribution because it is difficult to predict how many other memory requests are waiting to use the memory system. In the proposed model, we assume that at each state i would be an exponential server with average service time t_i_. we consider X to be a column vector such that each component x_i_ is the average time a memory request takes to finish using the memory system, assuming it started using the memory system at state i. First the memory request is served by state i, and on the average spends a time of x_i_ = (M^−1^e′)_i_. After that it either leaves memory systems or enters state j with probability p_ij_. It will spend on average time x_j_ to finally leave the memory system. Mathematically we have, in vector form,

1 $$\begin{aligned} {\text{X}} & = {\text{M}}^{{ - 1}} \varepsilon ^{\prime} + {\text{PX}} \\ {\text{X}} & = \left( {{\text{I}} - {\text{P}}} \right)^{{ - 1}} {\text{M}}^{{ - 1}} \varepsilon ^{\prime} = \left[ {{\text{M}}\left( {{\text{I }} - {\text{ P}}} \right)} \right]^{{ - 1}} \varepsilon ^{\prime} \\ \end{aligned}$$

Now, the random variable T denoting the time a memory request spends in memory system can be calculated as follows,

2 $$\text{T= }\sum_{\text{i=1}}^{\text{n}}{{\text{p}}_{\text{i}}{\text{x}}}_{\text{i}}= \text{ p X}$$

The rate matrix B is defined^23^ as,

3 $${\text{B}} = {\text{M}} \times \left( {{\text{I}} - {\text{P}}} \right)$$

The average visit time matrix is calculated as,

4 $${\text{V}} = {\text{B}}^{{ - {1}}}$$

The elements of the V matrix (v_ij_) represent the average time a memory request takes at state *j* from the time it first enters state *i* until it finishes using the memory system.

We can calculate the average service time of the memory system by taking the average of all states in the V matrix,

5 $${\text{E}}\left( T \right) = {\text{p}} \times {\text{V}} \times \varepsilon$$

From^23^ we know that the kth moment of the service time (T) is given by,

$${\text{E}}\left[ {{\text{T}}^{{\text{k}}} } \right] = {\text{k}}!{\text{p}} \times {\text{V}}^{{\text{k}}} \times \varepsilon$$

Which confirms the result in Eq. (5). The variance of the service time as in^23^,

6 $${\text{Variance }}\left( T \right) = {\text{E}}\left[ {{\text{T}}^{{2}} } \right]{-}\left( {{\text{E}}\left[ {\text{T}} \right]} \right)^{{2}} = {2}({\text{p}} \times {\text{V}} \times \varepsilon ){-}({\text{p}} \times {\text{V}} \times \varepsilon )^{{2}}$$

This model can be used as a baseline model since we assume there is no memory sharing. We can use this model for a specific system to calculate the values of the V matrix then from its values we will be able to indicate which level of the memory hierarchy represents the performance bottleneck. Hence, we can find for a specific application which level of memory needs to be enhanced to improve the system performance. Also, it can be used to measure the effect of sharing on the memory system performance. Finally, we can calculate the effect of the hit ratios and look up algorithms on the performance of existing computer systems. This model can be used for light loaded systems or for computer systems with a single customer.

### Concurrent access

**Figure 3:**
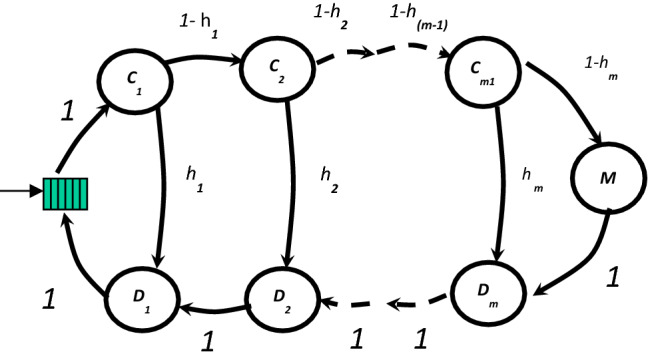
Markov chain for shared hierarchal memory system.

If concurrent access is allowed that means we can except more than one request to the memory hierarchy at the same time. If a request from one of the cores reaches the memory hierarchy when it is idle, it will be serviced immediately. If a request from one of the cores reaches the memory hierarchy when it is busy, it will wait in a FIFO queue until it gets its turn as shown in Fig. 3. Markov chains will not be adequate alone to model such system. Markov chains can be used to describe the different state of the memory hierarchy but cannot be used when we have a waiting queue. So, we will have to use a queueing model to describe our system. The distribution of the arrival time of memory requests can be either exponential or non-exponential. We assume that, for the proposed shared memory model, we can use the exponential distribution because it is difficult to predict how many other cores are waiting to use the memory system. For the service time distribution, we are going to use the non-exponential distribution since we are approximating the memory system with a Markov chain where each state is modeled as an exponential server. Based on the above assumptions, we use the M/G/1 model to represent the shared memory system in Fig. 3. By using the Pollaczek–Khintchine formula (P–K formula), we can estimate average number of memory requests waiting in the M/G/1 queue E(n) as follows,

7 $$\text{E(n)= }\frac{ \rho }{{1- \rho }}{+}\frac{{ \rho }^{2}}{1- \rho }{\times}\frac{{\text{C}}^{2}{-1}}{2}$$

The P–K formula is combined with Little’s theorem to show that the average memory system time E(T_s_) spent by a memory request in the M/G/1 queue is given by:

8 $${\text{E}}\left( {{\text{Ts}}} \right) = \frac{{{\text{E(n)}}}}{\lambda } = \frac{{{\text{E(T)}}}}{{1 - \rho }} + \frac{{{\text{E}}({\text{T}}) \times \rho }}{{1 - \rho }} \times \frac{{{\text{C}}^{2} - 1}}{2}$$

where λ is the average arrival rate of requests to the memory system, ρ is the utilization of the memory system, $\uprho \hspace{0.17em}=\hspace{0.17em}\uplambda \times \mathrm{ E}(\mathrm{T})$, C^2^ is the coefficient of variation of the memory access time, $\mathrm{C}\hspace{0.17em}=\hspace{0.17em}\mathrm{Variance }(\mathrm{T})/\mathrm{E}(\mathrm{T})^{2}.$

Note that, E (T) and Variance (T) can be calculated as we showed in the exclusive access case.

Previous work did not consider the variance of memory access times and its effect on the performance of shared memory systems. In Eq. (8), we estimate the average memory system time including the effect of the variance of the memory access time. Equation (8) shows that the average system time does not depend on the application type. We also differentiate between the memory response time (E (T)) and memory system time (E (T_s_)). The difference between the memory response time and memory system time represents the queueing delay for the memory system that has been ignored by previous models.

## Simulation results

**Figure 4:**
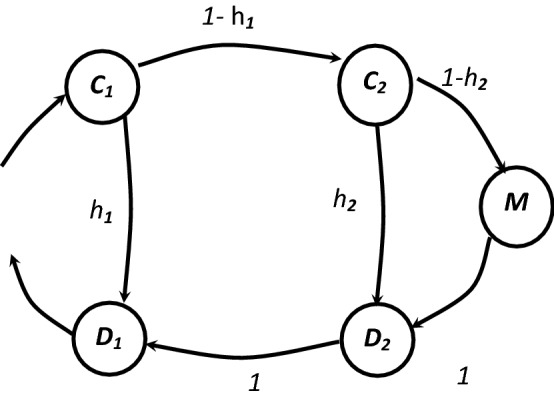
Markov chain for three hierarchal memory system.

We begin our parametric study by examining the effect of the system state on the performance of the memory system in exclusive memory access case. We use the proposed model to study the behavior of the memory hierarchy system of three levels. We will consider a memory system that consists of two levels of cache memory and one level of main memory as shown in Fig. 4. First, we need to construct the transition matrix and rate matrix using the different system parameters. The states of this system are,

C1 = the first level search process.

C2 = the second level search process.

M = accessing the main memory.

D1 = the first level read/write operation.

D2 = the second level read/write operation.

The stochastic Matrix P can be constructed as follows,

$$P= \left[\begin{array}{ccccc}0& 1-{h}_{1}& 0& 0& {h}_{1}\\ 0& 0& 1-{h}_{2}& {h}_{2}& 0\\ 0& 0& 0& 1& 0\\ 0& 0& 0& 0& 1\\ 0& 0& 0& 0& 0\end{array}\right]$$

The rate matrix M will be constructed as follows,

$$\mathrm{M}= \left[\begin{array}{ccccc}{\mathrm{M}}_{\mathrm{C}1}& 0& 0& 0& 0\\ 0& {\mathrm{M}}_{\mathrm{C}2}& 0& 0& 0\\ 0& 0& {\mathrm{M}}_{\mathrm{m}}& 0& 0\\ 0& 0& 0& {\mathrm{M}}_{\mathrm{D}2}& 0\\ 0& 0& 0& 0& {\mathrm{M}}_{\mathrm{D}1}\end{array}\right]$$

Here we will use the access time for the first level of cache T_D1_ as a one unit of time and we will use the rest of the time values according to this value as follows,

$$\begin{gathered} {\text{T}}_{{{\text{D1}}}} = :{\text{ access time for level one of the memory system}}. \hfill \\ {\text{T}}_{{{\text{D2}}}} = \, \gamma {\text{T}}_{{{\text{D1}}}} , \quad {\text{where }}\gamma > {1} \hfill \\ {\text{T}}_{{\text{m}}} = \, \gamma {\text{T}}_{{{\text{D2}}}} \hfill \\ {\text{T}}_{{{\text{C1}}}} = {\text{ T}}_{{{\text{C2}}}} = \, \alpha {\text{ T}}_{{{\text{D1}}}} , \quad {\text{where }}\alpha < {1} \hfill \\ \end{gathered}$$

The two parameters γ and α are used to reflect the cost and speed of the memory system. Small values of γ and α means a fast but more expensive system. The visit rate matrix is calculated as,

$$\mathrm{V }= {\mathrm{B}}^{-1}=\left[\begin{array}{ccccc}{\mathrm{T}}_{\mathrm{C}1}& {(1-{\mathrm{h}}_{1})\times\mathrm{T}}_{\mathrm{C}2}& (1- {\mathrm{h}}_{1})\times(1- {\mathrm{h}}_{2})\times{\mathrm{T}}_{\mathrm{m}}& (1-{\mathrm{h}}_{1})\times{\mathrm{T}}_{\mathrm{D}2}& {\mathrm{T}}_{\mathrm{D}1}\\ 0& {\mathrm{T}}_{\mathrm{C}2}& (1- {\mathrm{h}}_{2})\times{\mathrm{T}}_{\mathrm{m}}& {\mathrm{T}}_{\mathrm{D}2}& {\mathrm{T}}_{\mathrm{D}1}\\ 0& 0& {\mathrm{T}}_{\mathrm{m}}& {\mathrm{T}}_{\mathrm{D}2}& {\mathrm{T}}_{\mathrm{D}1}\\ 0& 0& 0& {\mathrm{T}}_{\mathrm{D}2}& {\mathrm{T}}_{\mathrm{D}1}\\ 0& 0& 0& 0& {\mathrm{T}}_{\mathrm{D}1}\end{array}\right]$$

The average service time is calculated as,

$$E(T)=\mathrm{p}\times \mathrm{V}\times\varepsilon$$

$$p = \left[\begin{array}{ccccc}1& 0& 0& 0& 0\end{array}\right]$$

**Figure 5:**
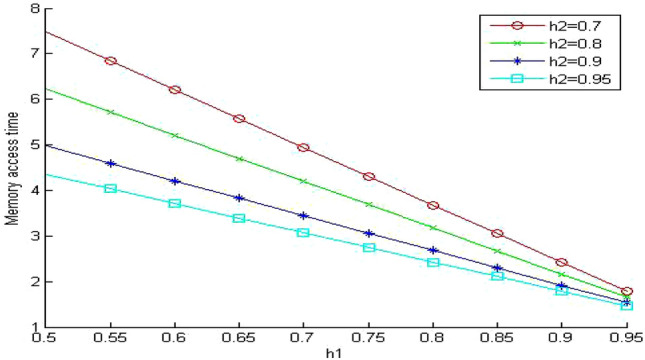
Average memory access time when γ = 5.

**Figure 6:**
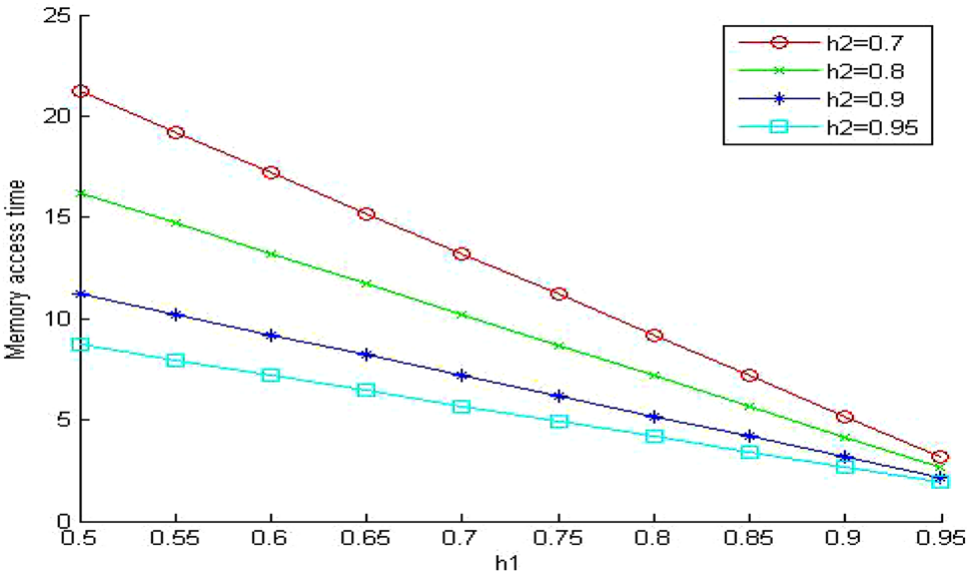
Average memory access time when γ = 10.

**Figure 7:**
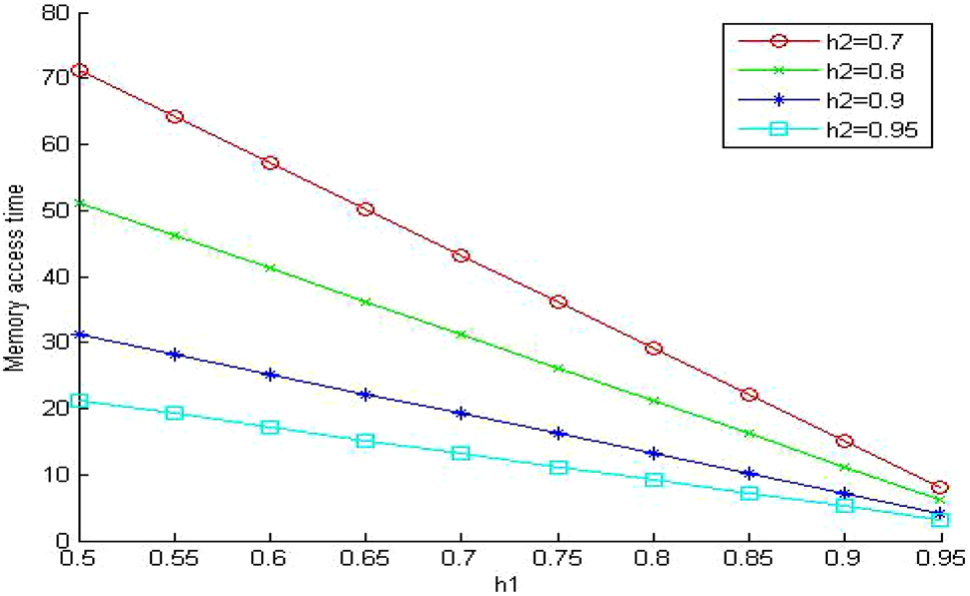
Average memory access time when γ = 20.

In Figs. 5, 6 and 7, we study the effect of changing the hit ratio of the second (h_2_) memory level on the total memory access time for different values of γ (5, 10, 20 respectively) when the hit ratio of the first memory level (h_1_) is constant. We can see clearly in these figures that the effect of h_2_ becomes more significant on the total memory hierarchy access time for large values of γ and small values of h_1_. For large values of h_1_ the effect of h_2_ on improving the total memory hierarchy access time increases for large values of γ. If we compare Figs. 5 and 7 when h_1_ equals 0.95, we see that the improvement of the total memory hierarchy access time approaches 20% when γ equals 5 and increases to 45% when γ equals 20.

**Figure 8:**
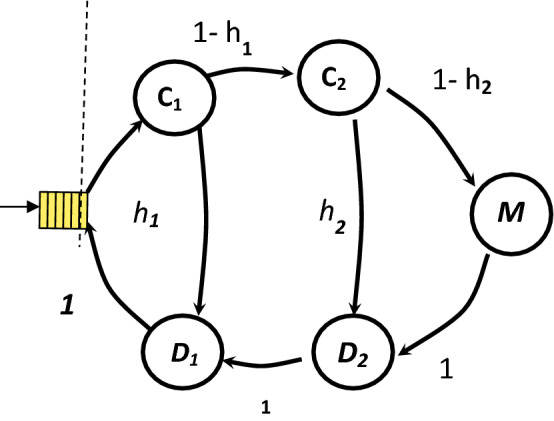
Markov chain for shared three hierarchal memory system.

Now we consider examining the effect of the system state on the performance of the memory system in the concurrent access case as in Fig. 8. We use the same parameters as in the exclusive memory access case to be able to estimate the effect of queueing. First, we assume that a light loaded system (ρ = 0.4) and the difference of access time between the memory levels is small (γ = 5 and α = 0.15).

**Figure 9:**
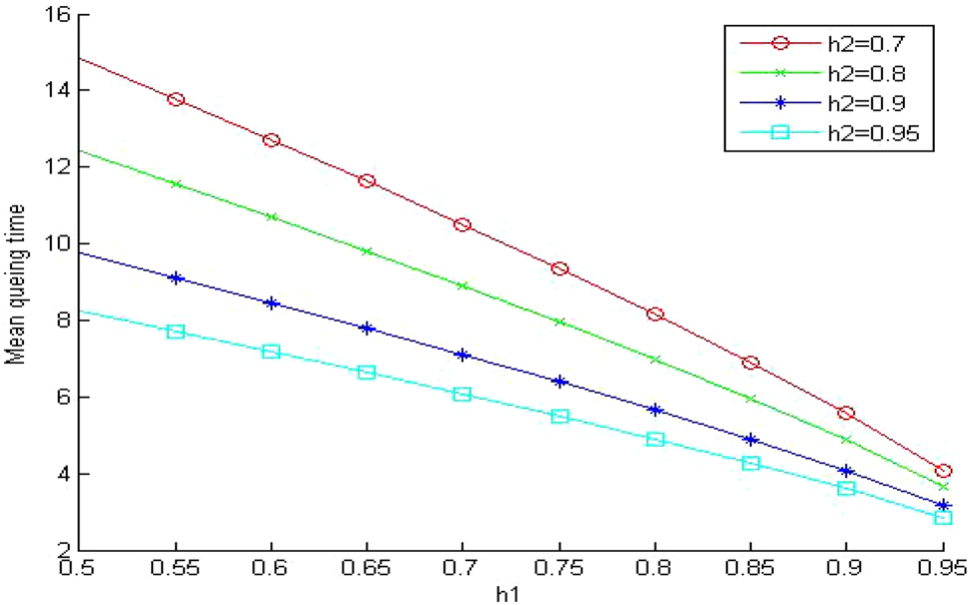
Mean memory system time when ρ = 0.4 and γ equals 5.

As we have seen in Fig. 5, the mean memory system time decreases as we increase h_1_. When we compare Fig. 9 with the similar system in Fig. 5, we can see clearly the effect of system sharing on the value of the memory system time. The values for the memory system time increased significantly (around 95%) even though the system is lightly loaded. The queuing effect becomes more significant for the small values of the hit ratios (h_1_ and h_2_). When we increase the values of the hit ratio over 0.9, the effect of the system sharing drops to 70% increase on average.

**Figure 10:**
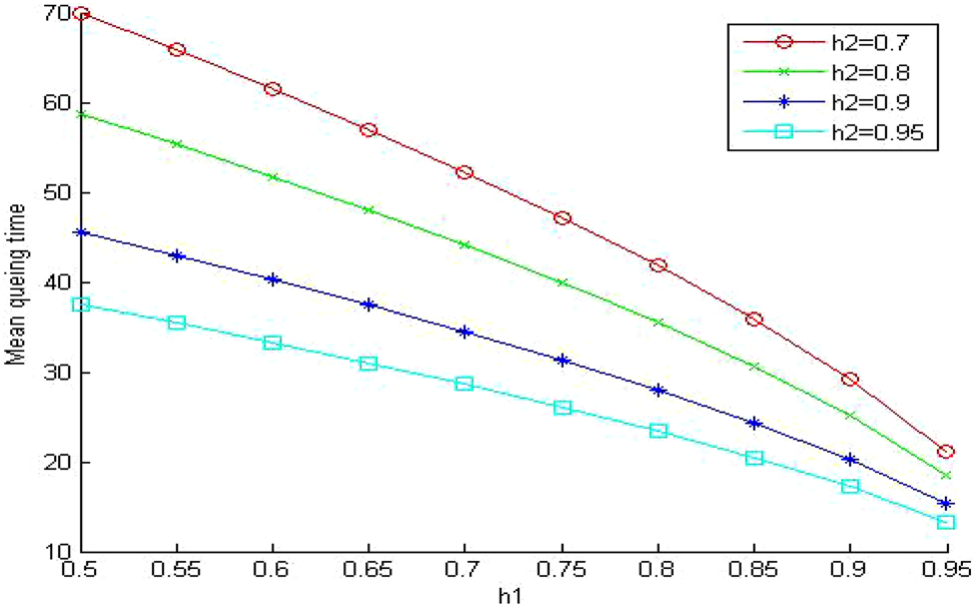
Mean memory system time when ρ = 0.85 and γ equals 5.

In Fig. 10, we increase the value of the memory system utilization to 0.85. Now the memory system is in a very busy state. We observe that the values of the memory system time increased to intolerable values. It reaches almost nine times the values of the similar system without queuing (Fig. 5). Surprisingly, the memory system time becomes even worse than the much cheaper system without queuing in (Fig. 7). The previous system is very expensive since the relative speed parameter between the different memory levels γ equals 5. The next step is to evaluate the impact of sharing on a less expensive system. We select the relative speed parameter γ to equal 20.

**Figure 11:**
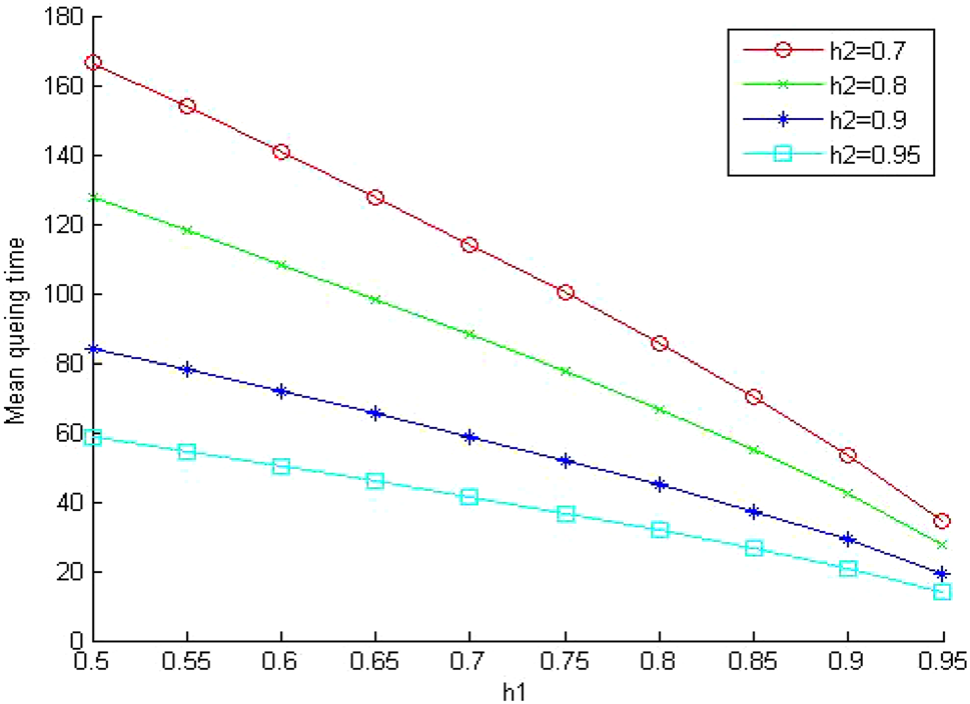
Mean memory system time when ρ = 0.4 and γ equals 20.

**Figure 12:**
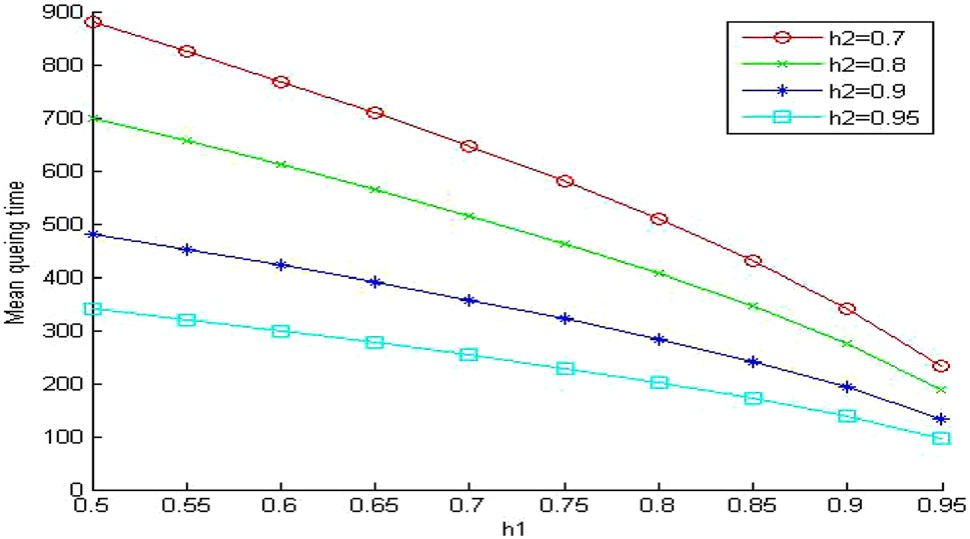
Mean memory system time when ρ = 0.85 and γ equals 20.

When we compare Figs. 11 and 7 (same system parameters without sharing), we can see that the effect of memory sharing has more influence on slow (cheap) system than fast (expensive) memory systems. The values for the memory system time increased significantly (approximately 130%) even though the system is lightly loaded. The increase in the memory system time for the fast system was (approximately 95%) as shown in Fig. 9. This influence increases significantly when we increase the memory system utilization ρ 0.85 as shown in Fig. 12.

None of the previous work studied the effect of the variance of the access time on the performance the memory hierarchy. It is well known that long tail distributions exist in many areas of computer systems such as FTP data connections, traffic arrivals in local and wide area networks, file sizes, and CPU burst times^24–26^. Even if we have a small value for memory access time, we could have a large variance if the distribution of memory access times has a long tail (coefficient of variation > 1). Memory access time large variance can produce long wait queues for the use of the shared memory systems that can significantly degrade the performance of multicore processors. A memory access time larger than a pipelined CPU depth can significantly influence the performance of pipelined processors because of the pipeline hazards and pipeline stalling^27^. The proposed model can take into account the dependency that may occur among the different memory hierarchy levels that influences the performance of the memory system.

**Figure 13:**
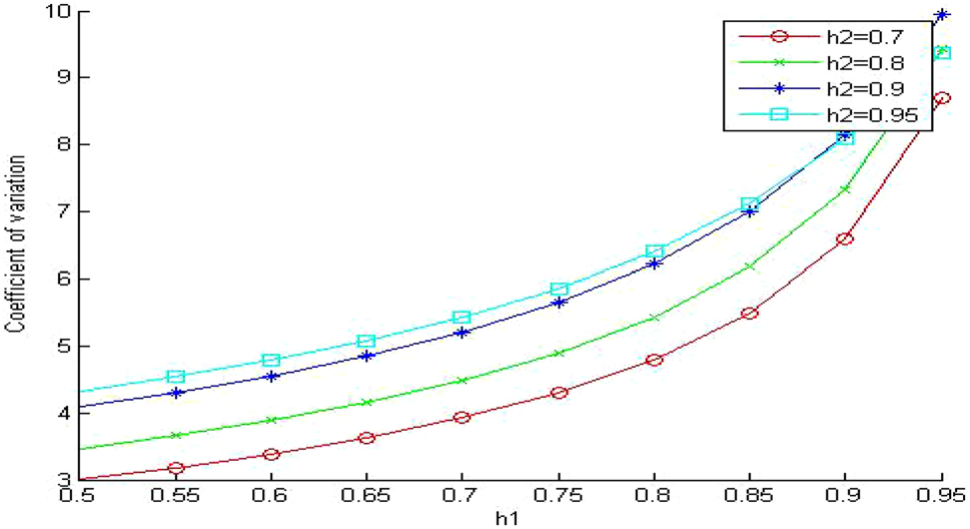
Coefficient of variation when γ = 20.

Next, we are going to study the effect of the memory hierarchy system on the coefficient of variation of memory access time. In Fig. 13, we show the effect of changing the value of the hit ratio of the first memory level (h_1_) on the coefficient of variation of memory access time. We use γ equals 20. As we can see, the coefficient of variation increases as we increase h_1_ but with different behavior for the different values of the hit ratios. For small values of the hit ratios, the coefficient of variation increases almost linearly. When the hit ratio h_1_ approaches 0.8, the coefficient of variation blows up.

**Figure 14:**
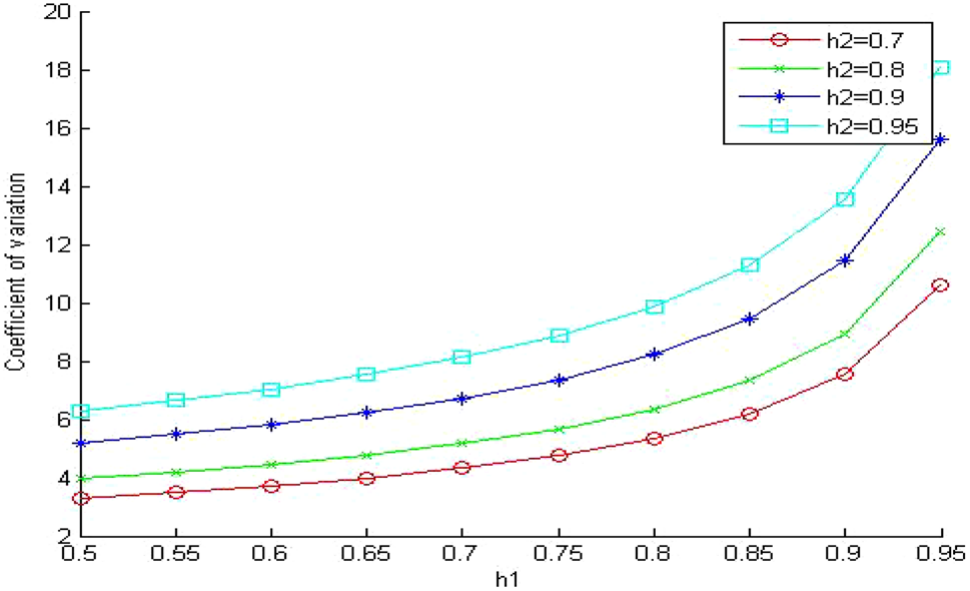
Coefficient of variation when γ = 50.

In Fig. 14, we show the effect of changing the hit ratio of the first memory level (h_1_) on coefficient of variation of memory access time when γ equals 50. We can notice the same behavior as in Fig. 13 except that the coefficient of variation increases more significantly. The values of the coefficient of variation increased by 80% for high values of h_1_ (greater than 0.8). This is a very important characteristic to consider when designing memory hierarchy systems. Since we always try to use memory levels with high hit ratios to improve the performance and this is true for single core processors. But for the memory hierarchy systems of multicore processors, we should consider the drawback of the queuing time that a memory request may face. The proposed model indicates that choosing memory levels with high hit ratios will improve the performance of memory hierarchy systems of multicore processors but with certain conditions.

- One of these conditions is to have a lightly loaded system. We should try to avoid building up the queue of the shared memory system. Otherwise the performance of the memory hierarchy system will degrade significantly.
- The other condition, is to have the relative speed between the memory levels (γ) as small as possible otherwise the variance of the memory response time may increase significantly.

## Conclusion

In this paper, we have proposed an analytical model based on Markov chains and the M/G/1 queueing model. The proposed analytical model can be used to model deep memory hierarchies for multicore processors. The main objective of the model is to evaluate the behavior of shared hierarchical memory systems by modeling their response time analytically. We have shown the impact of the number of hierarchical memory levels on the variance of the memory hierarchy response time. By using the proposed model, we were able to show that increasing the number of hierarchical levels of the memory increases the variance of the memory hierarchy response time. The large variance can produce long wait queues for the use of the shared memory systems which can significantly degrade the performance of multicore processors. The model can identify the performance bottlenecks in the hierarchal memory system by showing which level or levels of the memory hierarchy degrade the performance significantly. The model also can be used to estimate the waiting delay of the use of the shared memory systems. This delay explains the inconsistency between the values predicted by analytically models and the measured values published by previous researchers. We have shown that the waiting delay increases significantly when the relative speeds between memory levels increases.

## Data Availability

All data generated and analyzed during the current study are available from the corresponding author on reasonable request.

## References

[1] Djomehri, J., Jespersen, D., Taft, J., Jin, H., Hood, R. & Mehrotra, P. Performance of CFD applications on NASA supercomputers. In *Proceedings of the 21st Parallel Computational Fluid Dynamics Conference* (2009).

[2] Jin, H., Hood, R., Chang, J., Djomehri, J. & Jespersen, D. Characterizing application performance sensitivity to resource contention in multicore architectures NAS. Technical Report NAS-09-002. https://www.nas.nasa.gov/assets/pdf/techreports/2009/nas-09-002.pdf (2009).

[3] Field, D., Johnson, D., Mize, D. & Stober, R. Scheduling to overcome the multicore memory bandwidth bottleneck. HP Development Company, Technical Report (2007).

[4] Liu, M., Ji, W., Wang, Z., Li, J. & Pu, X. High performance memory management for a multicore architecture. In *9th IEEE International Conference on Computer and Information Technology* (2009).

[5] Berg, E. & Hagersten, E., StatCache: A probabilistic approach to efficient and accurate data locality analysis. In *IEEE International Symposium on Performance Analysis of Systems and Software* (2004).

[6] Eklov, D. & Hagersten, E. Efficient modeling of LRU caches. In *2010 IEEE International Symposium on Performance Analysis of Systems and Software* (2010).

[7] Pan, X. & Jonsson, B. A modeling framework for reuse distance-based estimation of cache performance. In *2015 IEEE International Symposium on Performance Analysis of Systems and Software* (2015).

[8] Ji K, Ling M, Zhang Y (2017). An artificial neural network model of LRU-cache misses on out-of-order embedded processors. J. Microprocess. Microsyst..

[9] Ji K, Ling M, Shi L (2017). Using the first-level cache stack distance histograms to predict multi-level LRU cache misses. J. Microprocess. Microsyst..

[10] Ji, K., Ling, M. & Liu, L. A probability model of calculating L2 cache misses. In *2018 International Conference on Computer Science, Electronics and Communication Engineering* (2018).

[11] Jasmine MS, Venkatesh TG (2018). Analytical miss rate calculation of L2 cache from the RD profile of L1 cache. IEEE Trans. Comput..

[12] Nikolov A (2008). Analytical model for a multiprocessor with private caches and shared memory. Int. J. Comput. Commun. Control.

[13] Oh, T., Lee, K. & Cho, S. An analytical performance model for co-management of last-level cache and bandwidth sharing. In *19th International Symposium on Modeling, Analysis and Simulation of Computer and Telecommunication Systems* (2011).

[14] Iyer, R. CQoS: A framework for enabling QoS in shared caches of CMP platforms. In *Proceedings of the 18th Annual International Conference on Supercomputing* (2004).

[15] Suh, G. E., Devadas, S. & Rudolph, L. A new memory monitoring scheme for memory-aware scheduling and partitioning. In *Proceeding of International Symposium of High-Performance Computer Architecture* (2002).

[16] Jin, R. & Agrawal, G. Performance prediction for random write reductions: A case study in modeling shared memory programs. In *Proceedings of the 2002 ACM SIGMETRICS International Conference on Measurement and Modeling of Computer Systems* (2002).

[17] Eklov, D., Black-Schaffer, D. & Hagersten, E. Fast modeling of shared caches in multicore systems. In *Proceedings of the 6th International Conference on High Performance and Embedded Architectures and Compilers* (2011).

[18] Jasmine MS, Venkatesh TG (2019). Analytical derivation of concurrent reuse distance profile for multi-threaded application running on chip multi-processor. IEEE Trans. Parallel Distrib. Syst..

[19] Wu, M. & Yeung, D. Coherent profiles: Enabling efficient reuse distance analysis of multicore scaling for loop-based parallel programs. In *Proceedings of the 2011 International Conference on Parallel Architectures and Compilation Techniques* (2011).

[20] Balasubramonian, R., Albonesiz, D., Buyuktosunoglu, A. & Sandhya, D. Dynamic memory hierarchy performance optimization. In *27th International Symposium on Computer Architecture* (2000).

[21] Xiaoqian L, Ming L, Guangmin W, Jiancong G (2021). Analytical modeling the multi-core shared cache behavior with considerations of data-sharing and coherence. IEEE Access.

[22] Serpa, M. *et al.* Memory performance and bottlenecks in multicore and GPU architectures. In *2019 27th Euromicro Conference on Parallel, Distributed and Network-Based Processing.*

[23] Lipsky L (2008). Queueing Theory—A Linear Algebraic Approach.

[24] Garg, S., Lipsky, L. & Robert, M. The effect of power-tail distribution on the behavior of time-sharing computer systems. In *ACM SIGAPP Symposium on Applied Computing* (1992).

[25] Lelan W, Taqqu M, Willinger W, Wilson D (1994). On the self-similar nature of ethernet traffic. IEEE/ACM Trans. Network..

[26] Paxson V, Floyd S (1995). Wide area traffic: The failure of Poisson Modeling. IEEE/ACM Trans. Network..

[27] Nanehkaran YA, SajjadBagheri B (2013). The challenges of multicore processor. Int. J. Adv. Res. Technol..

